# Pharmacoepigenetics of hypertension: genome-wide methylation analysis of responsiveness to four classes of antihypertensive drugs using a double-blind crossover study design

**DOI:** 10.1080/15592294.2022.2038418

**Published:** 2022-02-25

**Authors:** Marja-Liisa Nuotio, Heini Sánez Tähtisalo, Alexandra Lahtinen, Kati Donner, Frej Fyhrquist, Markus Perola, Kimmo K Kontula, Timo P Hiltunen

**Affiliations:** a Research Program for Clinical and Molecular Metabolism, Faculty of Medicine, University of Helsinki, Helsinki, Finland; b Department of Public Health Solutions, Finnish Institute for Health and Welfare (THL), Helsinki, Finland; c Department of Medicine, University of Helsinki and Helsinki University Hospital, Helsinki, Finland; d Research Program in Systems Oncology, Faculty of Medicine, University of Helsinki, Helsinki, Finland; e Technology Centre, Institute for Molecular Medicine Finland, University of Helsinki, Helsinki, Finland; f Minerva Foundation Institute for Medical Research, Helsinki, Finland

**Keywords:** Epigenomics, hypertension, pharmacogenetics, precision medicine

## Abstract

Essential hypertension remains the leading risk factor of global disease burden, but its treatment goals are often not met. We investigated whether DNA methylation is associated with antihypertensive responses to a diuretic, a beta-blocker, a calcium channel blocker or an angiotensin receptor antagonist. In addition, since we previously showed an SNP at the transcription start site (TSS) of the catecholamine biosynthesis-related *ACY3* gene to associate with blood pressure (BP) response to beta-blockers, we specifically analysed the association of methylation sites close to the *ACY3* TSS with BP responses to beta-blockers. We conducted an epigenome-wide association study between leukocyte DNA methylation and BP responses to antihypertensive monotherapies in two hypertensive Finnish cohorts: the GENRES (https://clinicaltrials.gov/ct2/show/NCT03276598; amlodipine 5 mg, bisoprolol 5 mg, hydrochlorothiazide 25 mg, or losartan 50 mg daily) and the LIFE-Fin studies (https://clinicaltrials.gov/ct2/show/NCT00338260; atenolol 50 mg or losartan 50 mg daily). The monotherapy groups consisted of approximately 200 individuals each. We identified 64 methylation sites to suggestively associate (P < 1E-5) with either systolic or diastolic BP responses to a particular study drug in GENRES. These associations did not replicate in LIFE-Fin . Three methylation sites close to the *ACY3* TSS were associated with systolic BP responses to bisoprolol in GENRES but not genome-wide significantly (P < 0.05). No robust associations between DNA methylation and BP responses to four different antihypertensive drugs were identified. However, the findings on the methylation sites close to the *ACY3* TSS may support the role of *ACY3* genetic and epigenetic variation in BP response to bisoprolol.

## Introduction

According to the recently published Global Burden of Disease Study 2019 carried out in over 200 countries, elevated systolic blood pressure (SBP) today occupies the globally leading position as a health risk factor, accounting for a total of 10.8 million deaths annually [1]. Furthermore, recent screening of adult subjects in 92 countries indicated that 34% had hypertension, of whom only 59% were aware of it and of whom 32% had hypertension under control (<140/90 mmHg) [2].

Poor individualization of antihypertensive drug treatment remains one possible cause for the disappointing results in reaching goals during management of hypertensive patients. Although a number of genome-wide pharmacogenomic studies have reported potential DNA markers for specific drug responses [3–9], there still appears to be lack of consistent findings to generate clinically meaningful predictive accuracy. Calculation of polygenic risk scores across approximately 900 independent genetic loci has been suggested to slightly improve risk stratification of hypertensive subjects [10], but initial attempts of their application to guide patient-specific antihypertensive drug treatment yielded only elusive data [11].

The fact that molecular genetic techniques have been able to detect only a small percentage of the postulated genetically determined portion of blood pressure (BP) variation has been called ‘missing heritability’ and has turned attention to gene alterations independent of sequence changes and epigenetic mechanisms in particular [12]. Epigenetic regulation of gene expression may take place through a variety of mechanisms, including DNA methylation, histone post-translational modification, and processing of non-coding RNAs [13–15].

To our knowledge, no pharmacoepigenomic studies of human hypertension, in which the extent of DNA methylation of genomic CpG sites is related to specific antihypertensive responses, have been carried out so far. We decided to use our pharmacogenomic GENRES (Genetics of Drug Responsiveness in Essential Hypertension) Study as a primary platform [5,16] for this purpose. In GENRES, the antihypertensive effects of four different drug classes (a diuretic, a beta blocker, a calcium channel blocker, and an angiotensin receptor antagonist) were studied in a placebo-controlled, rotational fashion, and DNA methylation analysis was carried out using the Illumina BeadChip system. We replicated the corresponding data on beta blocker and angiotensin receptor antagonist responses using DNA samples and BP data from the Finnish arm of the LIFE (Losartan Intervention for Endpoint Reduction in Hypertension) Study [17]. We have previously carried out genome-wide DNA association (GWAS) analyses for these two cohorts, thus also permitting comparison of epigenome-wide (EWAS) and GWAS data in genomic sites of interest.

## Materials and methods

### Study population

The overall design of the GENRES Study has been described in detail previously (https://clinicaltrials.gov/ct2/show/NCT03276598) [16]. In brief, it is a randomized, double-blind, placebo-controlled, cross-over study using four different antihypertensive monotherapies. The study subjects were 35- to 60-year-old men of Finnish ancestry and with moderate hypertension. The study protocol included a four-week initial wash-out placebo period, followed by four-week drug monotherapy periods (hydrochlorothiazide 25 mg, bisoprolol 5 mg, losartan 50 mg, and amlodipine 5 mg), separated by four-week placebo periods. Thus, the study subjects received each type of drug treatment in randomized order and are included in the analyses of all completed treatment periods. Measurements of office BP and 24-h ambulatory BP recordings were carried out after each drug and placebo period. The 24-h ambulatory BP recordings were performed using a device equipped with a QRS complex detector and a position sensor (Diasys Integra; Novacor, Rueil Malmaison, France). The single measurements in the ambulatory recordings were checked in a blinded fashion, and the 24-h BP levels were calculated as the mean of daytime (0700–2200 hours) and night-time (2200–0700 hours) values, weighed according to daytime and night-time hours, as described in detail earlier [16]. For the present EWAS study, we selected patients with imputed genotype data and ambulatory BP response data for at least one drug (198 for amlodipine, 204 for bisoprolol, 200 for hydrochlorothiazide, and 197 for losartan). The clinical part of the study was carried out in accordance with the Declaration of Helsinki and Guidelines for Good Clinical Practice (1996). The study was approved by the Ethics Committee of Helsinki University Hospital and the National Agency for Medicines of Finland. All screened subjects gave a signed informed consent prior to study activities.

The LIFE Study is an international, randomized, double-blind study originally aimed at evaluating the long-term treatment effects of losartan compared with atenolol in over 9 000 hypertensive patients with signs of left ventricular hypertension (https://clinicaltrials.gov/ct2/show/NCT00338260) [18]. After a 2-week placebo period, the patients were randomly assigned to receive either losartan (50 mg daily) or atenolol (50 mg daily). If the target BP (<140/90) was not reached at the two months’ visit, the treatment was intensified by the addition of hydrochlorothiazide and, at later visits, other antihypertensive drugs if needed. A pharmacogenetic substudy was performed in Scandinavia, including 1146 individuals of Finnish ancestry whose DNA samples were available for genetic studies [17]. Of this Finnish group, all 398 patients still on monotherapy at the two months’ visit (losartan, n = 198; atenolol, n = 200) were selected for EWAS analysis in the current study. Office BP measurements were recorded at baseline and after predetermined intervals during treatment. We used BP data recorded at the two months’ visit during monotherapy, comprising an average of two separate BP measurements. The main treatment protocol of the LIFE Study and the protocol of the genetic substudy were approved by the local ethics committees and performed in accordance with the Declaration of Helsinki. All participants gave a written informed consent before the study. Flow charts depicting the general design of the GENRES and the LIFE studies are illustrated in Supplementary Figure S1, and detailed characteristics of the study cohorts are presented in Table 1.

**Table 1.** General characteristics of the study cohorts, GENRES and LIFE-Fin.

	GENRES	LIFE-Fin
Total number of individuals (n)	219	398
Age (years)	50.5 ± 6.3	64 ± 6.2
Number of males (%)	219 (100%)	199 (50%)
Body mass index (kg/m^2^)	26.7 ± 2.7	27.4 ± 3.7
Number of current smokers (%)	32 (14.6%)	43 (10.8%)
Baseline SBP (mmHg)*	135 ± 10	166 ± 12
Baseline DBP (mmHg)*	93 ± 6	97 ± 6
**Blood pressure responses** Amlodipine		
ΔSBP (mmHg)	−7.4 ± 7.3	NA
ΔDBP (mmHg)	−4.9 ± 4.0	NA
Beta blocker**		
ΔSBP (mmHg)	−11.0 ± 5.9	−21.3 ± 12.7
ΔDBP (mmHg)	−8.3 ± 4.1	−12.8 ± 6.5
Hydrochlorothiazide		
ΔSBP (mmHg)	−4.8 ± 6.2	NA
ΔDBP (mmHg)	−1.7 ± 4.1	NA
Losartan		
ΔSBP (mmHg)	−9.0 ± 6.4	−21.0 ± 12.1
ΔDBP (mmHg)	−6.0 ± 4.5	−11.0 ± 7.0

Mean ± SD, or numbers and percentages are given. * 24 h-ambulatory BP values in GENRES and office BP values in LIFE-Fin. GENRES: amlodipine n = 198, bisoprolol n = 204, hydrochlorothiazide n = 200, and losartan n = 195. LIFE-Fin: atenolol n = 200 and losartan n = 197. ** Bisoprolol in GENRES and Atenolol in LIFE-Fin. Δ = change, SBP = systolic blood pressure, DBP = diastolic blood pressure

### DNA methylation profiling

DNA was extracted from peripheral blood leukocytes using standard methods. In the GENRES Study, more than 95% of the blood samples for methylation analysis were drawn before the clinical study, whilst in LIFE, the blood samples were drawn before or during the clinical study. Only one DNA sample per patient was analysed. Genome-wide methylation profiling was performed at the Institute for Molecular Medicine Finland (FIMM) Technology Centre, University of Helsinki. Bisulphite conversion of 1 µg of DNA was performed using the EZ-96 DNA Methylation Kit (Zymo Research, Irvine, CA, USA), according to the manufacturer’s instructions. Four µl of bisulphite-converted DNA was whole-genome amplified, enzymatically fragmented, and hybridized to the Infinium MethylationEPIC_v-1-0 BeadChip (Illumina, San Diego, CA, USA) according to the manufacturer’s protocol. The BeadChips were scanned using an iScan reader (Illumina).

### Infinium methylation EPIC BeadChip methylation measurements

Methylation data preprocessing and quality control were performed as described in the R package ‘minfi’ v1.18.4 [19], using R software v3.6.1 (R Core Team, 2016) [20]. First, we preprocessed the raw intensity data files and generated M-values (log2 ratio of the intensities of the methylated probe vs unmethylated probe). We next corrected the methylation values for background and applied normalization with a subset quantile normalization approach (SWAN) [21]. The inspection of the control probe signals revealed no outliers and no sex discrepancies after checking the sex prediction. The quality control steps included the removal of the probes with a low (< 95%) detection rate at P values < 0.01 and probes located on sex chromosomes. As outlined in the study by Chen et al [22], we excluded known cross-reactive probes and probes containing a single-nucleotide polymorphism either at the CpG interrogation or at the single-nucleotide extension. After the quality control steps, 808,832 and 812,839 CpG (for GENRES and LIFE-Fin, respectively) probes were used for the epigenome-wide analyses. One sample from the GENRES study had less than 800,000 detected CpG sites and was excluded from the analyses.

### Pathway analysis

Enriched pathways were explored using Enrichr (https://maayanlab.cloud/Enrichr/) [23–25], in particular Kyoto Encyclopedia of Genes and Genomes (KEGG) 2021 Human and WikiPathway 2021 Human pathway libraries. A list of genes (20 and more genes) corresponding to the most significant EWAS results for BP responses to antihypertensive drugs in GENRES (Table 2, ‘Nearest gene,’ separately for bisoprolol and losartan) served as the input. The Benjamini-Hochberg (BH) procedure was used to correct for multiple testing, and BH-adjusted P-values < 0.05 and pathways with two or more genes were considered statistically significant.

**Table 2.** The most significant (P < 10^−5^) EWAS results for blood pressure responses to antihypertensive drugs in GENRES. Ranked by the P value.

					ΔSBP			ΔDBP	
		CpG	Chr: position	Nearest gene	Effect	P-value		Effect	P-value
**Amlodipine**									
		cg22168795	17: 34 463 933	*CCL4*	*	*		1.5	4.0 × 10^−6^
		cg09664259	3: 156 544 070	*LEKR1*	−2.3	5.1 × 10^−6^		*	*
		cg17854544	5: 141 344 968	*RNF14*	−3.1	7.0 × 10^−6^		*	*
**Bisoprolol**									
	cg14158424		9 126 763 957	*LHX2*	*	*		−2.0	5.2 × 10^−7^
	cg05560731		9: 115 632 737	*SNX30*	3.0	4.6 × 10^−6^		2.2	8.4 × 10^−7^
	cg21740631		5: 167 660 070	*TENM2*	*	*		2.8	8.8 × 10^−7^
	cg01938422		1: 35 659 480	*SFPQ*	2.6	1.7 × 10^−6^		*	*
	cg18012642		6: 119 255 579	*MCM9*	−2.7	2.5 × 10^−6^		*	*
	cg05347334		4: 2 439 397	*RP11-503N18.1*	*	*		1.6	2.8 × 10^−6^
	cg12667196		9: 108 456 734	*TMEM38B*	−2.6	3.4 × 10^−6^		−1.8	9.5 × 10^−6^
	cg14275626		9: 135 549 588	*GTF3C4*	*	*		3.2	3.6 × 10^−6^
	cg04640885		2: 145 273 345	*ZEB2*	*	*		−1.7	3.9 × 10^−6^
	cg23054533		8: 139 095 979	*RP11-238K6.1*	*	*		1.6	4.4 × 10^−6^
	cg16866321		7: 62 153 290	*RP11-196D18.1*	2.2	5.4 × 10^−6^		*	*
	cg04822851		1: 203 095 988	*ADORA1*	−2.1	5.9 × 10^−6^		*	*
	cg13889422		2: 220 492 557	*SLC4A3*	−2.6	5.9 × 10^−6^		*	*
	cg19703259		16: 70 612 843	*IL34*	3.7	5.9 × 10^−6^		*	*
	cg07021268		19: 47 921 051	*MEIS3*	*	*		−1.5	6.9 × 10^−6^
	cg19755776		6: 29 067 386	*SERPINB6*	2.7	7.0 × 10^−6^		*	*
	cg01074392		21: 44 037 324	*AP001626.1*	−2.4	7.6 × 10^−6^		*	*
	cg13097433		17: 42 877 358	*GJC1*	*	*		1.7	7.7 × 10^−6^
	cg11706030		2: 163 225 840	*GCA; KCNH7*	*	*		1.6	7.7 × 10^−6^
	cg06206086		1: 36 412 029	*AGO3*	2.1	8.0 × 10^−6^		*	*
	cg03228312		20: 50 808 336	*ZFP64*	−2.8	8.6 × 10^−6^		*	*
	cg21541833		2: 207 507 096	*AC010731.4*	−2.5	8.8 × 10^−6^		*	*
	cg22065976		6: 33 589 061	*ITPR3*	*	*		−1.9	9.0 × 10^−6^
**Hydrochlorothiazide**									
	cg21240861		7: 157 129 652	*DNAJB6*	−2.6	2.1 × 10^−6^		−1.8	2.1 × 10^−6^
	cg07851807		1: 44 024 223	*PTPRF*	*	*		−1.8	4.9 × 10^−6^
	cg15555606		8: 56 280 194	*XKR4*	−2.8	5.0 × 10^−6^		*	*
	cg00577969		11: 122 156 457	*RP11-716H6.1*	−2.3	5.2 × 10^−6^		*	*
	cg14779520		5: 14 696 254	*FAM105B*	*	*		−1.6	8.3 × 10^−6^
	cg00407040		10: 14 247 935	*FRMD4A*	−2.5	8.7 × 10^−6^		*	*
**Losartan**									
	cg14496951		1: 156 265 517	*GLMP*	−3.4		9.0 × 10^−8^	*	*
	cg14994060		5: 134 376 489	*C5orf66*	−3.5		6.7 × 10^−7^	*	*
	cg19782883		1: 210 406 123	*SERTAD4*	−3.3		8.4 × 10^−7^	*	*
	cg14745622		20: 61 447 686	*COL9A3*	−3.6		9.2 × 10^−7^	*	*
	cg05014952		14: 35 873 130	*NFKBIA*	−3.7		1.4 × 10^−6^	*	*
	cg25955837		6: 46 620 788	*SLC25A27; CYP39A1*	−3.5		1.4 × 10^−6^	*	*
	cg11621667		7: 39 662 995	*RALA*	−3.8		1.8 × 10^−6^	*	*
	cg04640216		11: 63 439 065	*ATL3*	*		*	−1.9	2.0 × 10^−6^
	cg00322946		10: 22 605 631	*COMMD3*	−3.3		2.0 × 10^−6^	*	*
	cg00383296		4: 42 400 551	*SHISA3*	−3.1		2.3 × 10^−6^	*	*
	cg08681519		22: 41 810 229	*TEF*	−3.3		3.1 × 10^−6^	*	*
	cg02181494		22: 51 066 755	*ARSA*	−3.0		3.2 × 10^−6^	*	*
	cg27270003		4: 37 828 093	*PGM2*	−2.9		3.5 × 10^−6^	*	*
	cg17712828		10: 94 833 632	*CYP26A1*	−3.1		4.3 × 10^−6^	*	*
	cg01653417		17: 80 256 028	*HES7*	−3.6		4.9 × 10^−6^	*	*
	cg07922719		9: 117 150 338	*AKNA*	−3.0		4.9 × 10^−6^	*	*
	cg12446722		1: 226 374 380	*ACBD3*	−4.4		5.4 × 10^−6^	*	*
	cg20458560		6: 146 283 629	*SHPRH*	4.0		5.7 × 10^−6^	*	*
	cg27204776		2: 203 777 060	*CARF;WDR12*	−4.3		6.4 × 10^−6^	*	*
	cg20250570		10: 1 034 318	*GTPBP4*	−3.3		6.5 × 10^−6^	*	*
	cg15147060		3: 88 108 213	*CGGBP1*	−3.7		6.7 × 10^−6^	*	*
	cg26326168		12: 133 405 726	*CHFR; GOLGA3*	−3.0		6.8 × 10^−6^	*	*
	cg07485279		3: 31 574 058	*STT3B*	−2.9		7.0 × 10^−6^	*	*
	cg10014408		9: 139 305 226	*PMPCA;SDCCAG3*	−2.6		7.2 × 10^−6^	*	*
	cg06637893		16: 67 700 960	*C16orf86;ENKD1*	−3.1		7.6 × 10^−6^	*	*
	cg06323912		16: 57 481 690	*CIAPIN1;COQ9*	−3.4		7.7 × 10^−6^	*	*
	cg00876175		3: 179 615 032	*PEX5L*	3.7		8.4 × 10^−6^	*	*
	cg14851297		11: 35 965 488	*LDLRAD3*	−2.8		9.0 × 10^−6^	*	*
	cg10007405		13: 19 174 773	*LINC00388*	2.6		9.1 × 10^−6^	*	*
	cg04107773		20: 62 273 555	*STMN3*	2.9		9.7 × 10^−6^	*	*
	cg27438067		10: 2 119 638	*RP11-69C17.2*	2.9		9.7 × 10^−6^	*	*

The physical positions are given as the Genome Reference Consortium human genome build 37 coordinates.

*P-value >10^−5^. CpG = cytosine–guanine dinucleotide, Chr = chromosome, Δ = change, SBP = systolic blood pressure, DBP = diastolic blood pressure.

### ACY3 *methylation analysis*

As part of the *ACY3* methylation analysis, we analysed the association of the *ACY3* single-nucleotide polymorphism (SNP) rs2514036 with the methylation degree of its closest methylation sites (mQTL, i.e., methylation quantitative trait locus analysis) using linear regression, where age, sex (in LIFE-Fin), smoking status, alcohol consumption, and cell types were used as covariates, and slide and array as fixed factors.

### Statistical analyses of the epigenome-wide analysis

In the epigenome-wide association analyses of the GENRES and LIFE-Fin cohorts, the association between the degree of methylation (M-values) of CpG sites and BP responses to four different drugs were tested using a regression analysis fitting generalized linear model (*glm*). *R* Statistical software program version 3.2.0 was used to perform all statistical analyses (R Core Team, 2016) [20]. The R script for the linear regression analysis is included in Supplementary Methods. In GENRES, the SBP and diastolic blood pressure (DBP) responses of the study drugs were calculated as the change in 24-hour ambulatory BP values; the means of all (up to four) placebo periods were used as the baseline levels. Two GENRES subjects with BP responses deviating more than 4 standard deviations from the mean were excluded from the analyses. In LIFE-Fin, the BP responses were calculated as the change in office BP values after two months’ treatment using the values after the 2-week run-in placebo period as the baseline levels (there were no outliers).

In GENRES, the analyses were adjusted for age, baseline BP (systolic or diastolic, according to the dependent variable), smoking status (defined as current smokers/non-smokers), alcohol consumption, body mass index, serum creatinine, genetic principal components 1 to 3 and six cell types (the relative proportions of cell types in whole blood estimated using the statistical method described by Houseman et al. [26]). Additionally, technical parameters of the slide and chip array were used as covariates to address batch variance. The principal components were generated as described earlier [27]. In LIFE-Fin, a similar analytical model was used with the exception of including sex amongst the covariates. Non-normally distributed variables in GENRES (methylation degree, baseline SBP, and genetic principal component 2) were normalized with Blom’s transformation prior to analyses. In LIFE-Fin, normalized variables included the methylation degree and genetic principal component 2. In GENRES, alcohol consumption was classified into five categories by days of alcohol drinking per month: non-drinkers, 1 to 5 days per month (d/mo), 6 to 10 d/mo, 11 to 20 d/mo, and over 20 d/mo. In LIFE-Fin, alcohol intake was classified into five categories by alcohol intake per week (doses/wk): non-drinkers, 1 to 4 doses/wk, 5 to 7 doses/wk, 8 to 10 doses/wk, and over 10 doses/wk.

In the analysis of discovery EWAS data, P-values < 5 × 10^−8^ were considered statistically significant and P-values <10^−5^ statistically suggestively significant.

## Results

### General characteristics of the study cohorts, GENRES and LIFE-Fin

Table 1 summarizes the relevant clinical characteristics and antihypertensive drug responses of the GENRES and LIFE-Fin patients. The GENRES Study comprised men only, whilst in the LIFE-Fin Study, an equal number of women and men participated. The different study designs are also reflected in the different baseline BP levels and BP responses: the GENRES Study included only moderately hypertensive middle-aged subjects without significant echocardiographic left ventricular hypertrophy, whilst the subjects of the LIFE-Fin Study were older and had signs of electrocardiographic left ventricular hypertrophy. In addition, in GENRES, the baseline BP values are derived from ambulatory 24-h recordings after up to four placebo periods, whilst only office BP measurements after a two-week placebo period were available for the LIFE-Fin subjects.

### Epigenome-wide association between DNA methylation and blood pressure responses to antihypertensive drugs

We carried out an EWAS across the 808,832 and 812,839 (in GENRES and LIFE-Fin, respectively) methylation sites and BP responses to the four different antihypertensive drugs, including amlodipine, bisoprolol, hydrochlorothiazide, and losartan, in 197–204 individuals in GENRES. As shown in Table 2, we identified 63 different methylation sites that are suggestively associated (P < 10^−5^) with either SBP or DBP change caused by a given study drug. An effect size of 1 corresponds to a 1-mmHg difference in BP response per 1 SD of normalized methylation degree. None of the examined methylation sites showed simultaneous association (P < 10^−5^) with two or more of the study drugs. Of the identified methylation sites, 3 were associated with BP response to amlodipine and corresponding associations were recorded for 23 sites to bisoprolol, 6 sites to hydrochlorothiazide, and 31 sites to losartan. QQ plots for the drug-specific regression model results (P-values) of BP and methylation degree associations in GENRES and corresponding lambdas showed no significant test-statistic inflation. This is illustrated in Supplementary Figure S2, which displays the association tests for BP responses to different antihypertensive drugs.

Most of the identified methylation sites were associated (P < 10^−5^) with either SBP or DBP, with seven associations reaching a significance level of P < 10^−6^. Cg14158424 in LHX2 on chromosome 9, cg05560731 in *SNX30* also on chromosome 9, and cg21740631 in *TENM2* located on chromosome 5 showed association with the change in DBP by bisoprolol (β = −2.0, P = 5.2 × 10^−7^; β = 2.2, P = 8.4 × 10^−7^; and β = 2.8, P = 8.8 × 10^−7^, respectively). Cg14496951 located in an immediate proximity of *GLMP* on chromosome 1, cg14994060 on chromosome 5, cg19782883 in *SERTAD4* located on chromosome 1, and cg14745622 in *COL9A3* located on chromosome 20 showed associations with the change in SBP by losartan (β = −3.4, P = 9.0 × 10^−8^; β = −3.5, P = 6.7 × 10^−7^; β = −3.3, P = 8.4 × 10^−7^; and β = −3.6, P = 9.2 × 10^−7^, respectively).

**Figure 1.**
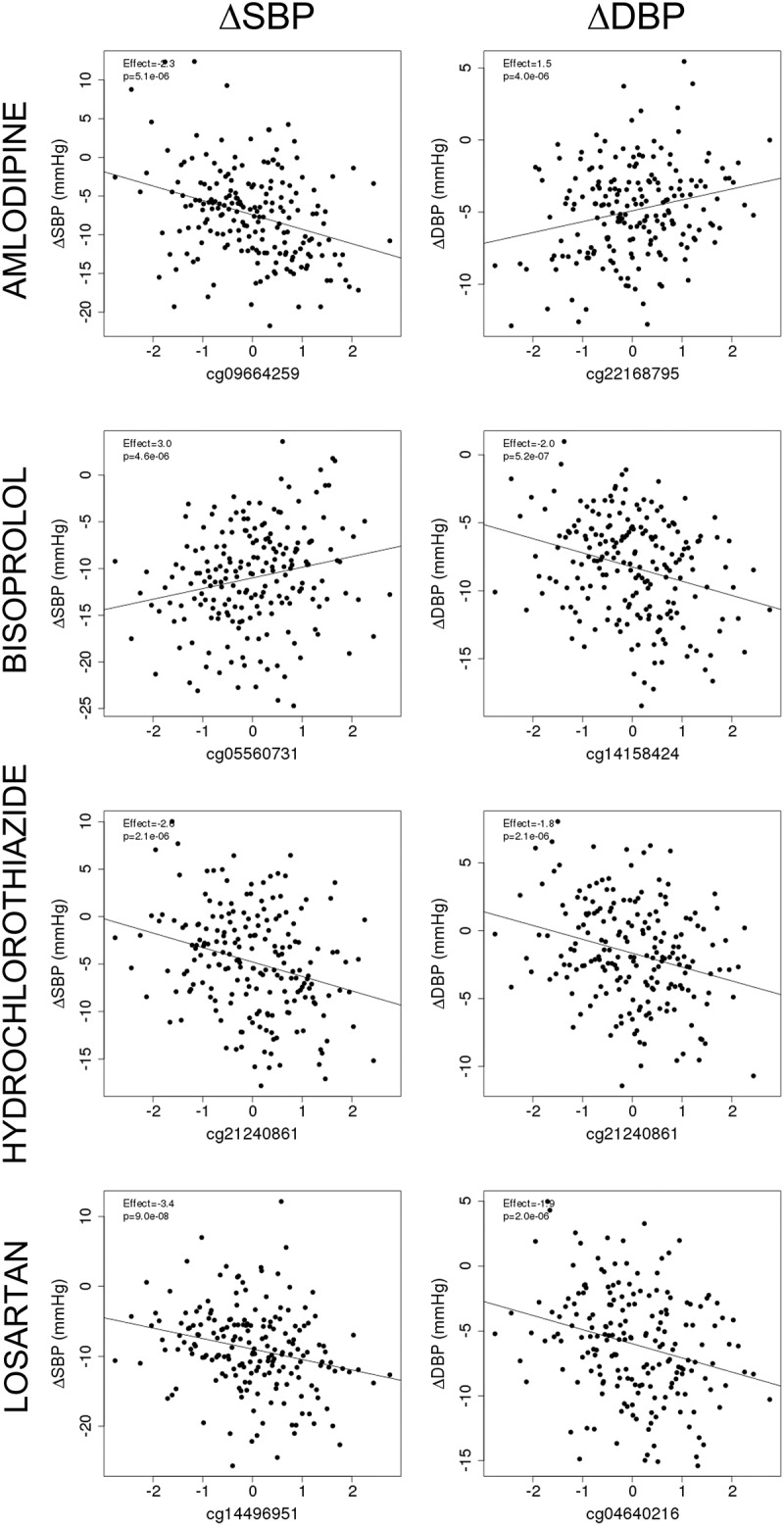
**Scatter plots for the most significant correlations between normalized methylation degrees (M-values) and covariate-adjusted blood pressure responses in GENRES**. Results from the EWAS analysis are shown as effect sizes and P values. A linear regression line is displayed.

Two individual sites were associated with both SBP and DBP responses, one for bisoprolol (cg05560731 in *SNX30* on chromosome 9, β = 3.0, P = 4.6 × 10^−6^ for SBP and β = 2.2, P = 8.4 × 10^−7^ for DBP) and the other for hydrochlorothiazide (cg21240861 in *DNAJB6* on chromosome 7, β = −2.6, P = 2.1 × 10^−6^ for SBP and β = −1.8, P = 2.1 × 10^−6^ for DBP). We present drug-specific correlation scatter plots for the most significant associations of methylation degrees with BP responses in GENRES (Figure 1). We analysed whether the most significant (P < 10^−5^) associations for bisoprolol and losartan BP responses replicated in the LIFE-Fin cohort for atenolol and losartan, respectively. However, these associations did not replicate on a statistically significant level (see Supplementary Table S1, which illustrates the results of the replication analyses in LIFE-Fin). When only males of the LIFE-Fin cohort were analysed, the results remained non-significant (data not shown).

### Pathway analysis for the top results from the epigenome-wide association study

To explore the potentially affected biological pathways, we subjected the sets of genes resulted from EWAS for bisoprolol (24 genes) and losartan (37 genes) to pathway analyses using Enrichr [23–25]. The gene sets for amlodipine (3 genes) and hydrochlorothiazide (6 genes) were not used for pathway analyses due to a limited number of genes.

In the case of the bisoprolol gene set, the top findings included three separate pathways. The first was KEGG 2021 Human pathway ‘Renin secretion’ with the significance level of P = 0.003 (unadjusted, BH-adjusted P = 0.179). Corresponding genes of this pathway were found to be *ADORA1* and *ITPR3*. The second was the WikiPathway 2021 Human pathway ‘Calcium regulation in the cardiac cell WP536’ (unadjusted P = 0.014, BH-adjusted P-value = 0.069, corresponding genes *GJC1* and *ITPR3*). The third was the ‘Circadian rhythm-related genes WP536’ pathway (unadjusted P = 0.002, BH-adjusted P = 0.042, corresponding genes *ADORA1, SFPQ*, and *KCNH7*).

For the losartan gene set, the top findings included WikiPathway 2021 Human ‘Oxidation by cytochrome P450 WP43’ (unadjusted P = 0.006, BH-adjusted P = 0.156, corresponding genes *CYP39A1* and *CYP26A1*).

### *Methylation sites across the* ACY3 *promoter regions were associated with blood pressure responses to beta blockers*

**Figure 2.**
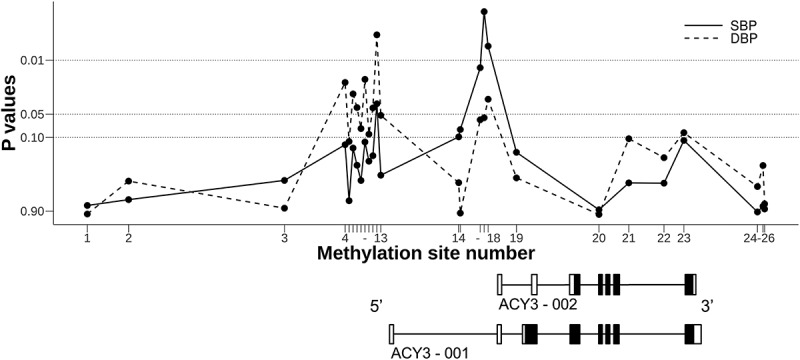
**P-values for the associations of methylation sites across *ACY3* with blood pressure response to bisoprolol in the GENRES Study**. Black boxes on the transcript schemes represent coding exons, and white boxes non-coding exons. Lines between boxes depict introns. For methylation (CpG) site numbering (on the abscissa) and precise genomic positioning, see Table 3

*ACY3* on chromosome 11 encodes two different transcripts, ACY3-001 and ACY3-002 (see Figure 2). *ACY3* codes for aminoacylase 3 that plays a potential role in catecholamine metabolism [28]. In view of our previous finding demonstrating an association between genetic variation (rs2514036) at the ACY3-002 transcription start site (TSS) and BP response to beta blockers [17], we were interested in possible associations between methylation sites close to *ACY3* TSSs and beta blocker responses in the same individuals.

In GENRES, none of the observed associations met the genome-wide or suggestive significance levels. The most significant associations were found between BP responses to bisoprolol and methylation sites located close to (mostly upstream) the TSSs of ACY3-001 and ACY3-002 (Table 3). The methylation sites close to the TSS of ACY-002 (corresponding to CpG numbers 16 to 18 in Table 3) were associated with both SBP and DBP responses to bisoprolol (P-values from 0.002 to 0.06). In LIFE-Fin, corresponding associations were observed for the SBP responses to atenolol with a P-value of 0.03 for the CpG number 16. The methylation sites close to the TSS of ACY-001 (CpG numbers 4 to 13) were associated with mainly DBP responses to bisoprolol in GENRES (P-values from 0.005 to 0.11). Clustering of the lowest P-values around the two TSSs is schematically illustrated in Figure 2.

**Table 3.** Associations of methylation sites across the *ACY3* promoter regions with blood pressure responses to beta blockers.

					Bisoprolol (GENRES)*				Atenolol (LIFE-Fin)*			
					ΔSBP		ΔDBP		ΔSBP		ΔDBP	
No	CpG	Position on chr 11	Relative distance (bp) to ACY3-001 TSS#	Relative distance (bp) to ACY3-002 TSS#	Effect	P-value	Effect	P-value	Effect	P-value	Effect	P-value
1	cg12371216	67 425 188	−7 058	−10 134	0.2	0.76	0.01	0.98	−0.8	0.55	−0.2	0.72
2	cg19551037	67 424 148	−6 018	−9 094	−0.2	0.64	0.3	0.37	−0.1	0.93	−0.4	0.41
3	cg01549428	67 420 220	−2 090	−5 166	−0.5	0.36	−0.1	0.82	0.3	0.79	−0.2	0.72
4	cg23130075	67 418 365	−235	−3 311	−0.8	0.12	−0.8	0.02	−1.0	0.37	−0.3	0.51
5	cg04986336	67 418 358	−228	−3 304	−0.3	0.66	−0.6	0.11	−2.0	0.08	−0.3	0.58
6	cg12355172	67 418 315	−185	−3 261	−0.8	0.14	−0.8	0.03	−0.9	0.41	−0.2	0.77
7	cg09296957	67 418 310	−180	−3 256	−0.6	0.23	−0.7	0.04	−1.7	0.13	−0.1	0.80
8	cg25835179	67 418 291	−161	−3 237	−0.5	0.36	−0.6	0.08	−1.5	0.17	−0.3	0.53
9	cg00350199	67 418 213	−83	−3 159	−0.8	0.11	−0.9	0.02	−0.6	0.59	−0.2	0.67
10	cg06148175	67 418 148	−18	−3 094	−0.6	0.20	−0.6	0.09	−1.0	0.34	−0.3	0.51
11	cg04565008	67 418 140	−10	−3 086	−0.7	0.17	−0.7	0.04	−1.5	0.17	−0.3	0.53
12	cg01240599	67 418 045	85	−2 991	−1.1	0.04	−1.1	0.005	−1.6	0.14	−0.1	0.82
13	cg11096993	67 417 958	172	−2 904	−0.5	0.31	−0.7	0.05	−1.1	0.33	−0.4	0.47
14	cg15800282	67 415 829	2 301	−775	1.3	0.10	0.5	0.39	−1.5	0.40	−1.5	0.08
15	cg10254998	67 415 788	2 342	−734	1.4	0.08	−0.03	0.95	−4.9	0.008	−1.7	0.07
16	cg05214293	67 415 194	2 936	−140	−2.0	0.013	−1.1	0.06	−4.6	0.03	0.3	0.74
17	cg05849431	67 415 187	2 943	−133	−1.9	0.002	−0.9	0.06	−1.7	0.27	0.2	0.78
18	cg07252184	67 415 183	2 947	−129	−1.8	0.007	−1.0	0.03	−1.8	0.24	0.1	0.90
19	cg23879118	67 414 373	3 757	681	−0.8	0.16	−0.4	0.33	−1.1	0.30	0.2	0.65
20	cg00793342	67 412 293	5 837	2 761	0.1	0.86	−0.004	0.99	−0.3	0.87	0.6	0.45
21	cg11964549	67 411 541	6 589	3 513	−0.5	0.39	−0.6	0.10	−0.9	0.45	−0.1	0.90
22	cg16320405	67 410 654	7 476	4 400	−0.4	0.39	−0.5	0.18	0.4	0.67	0.0	0.99
23	cg03933203	67 410 153	7 977	4 901	1.0	0.11	0.8	0.09	−1.4	0.45	−1.0	0.29
24	cg03889015	67 408 299	9 831	6 755	−0.1	0.92	−0.4	0.43	0.8	0.59	−1.3	0.05
25	cg05357287	67 408 159	9 971	6 895	0.2	0.78	0.6	0.23	−0.6	0.68	−1.2	0.09
26	cg00536203	67 408 119	10 011	6 935	−0.1	0.84	−0.2	0.73	0.1	0.92	−0.7	0.31

The CpG sites are listed in reverse order since *ACY3* is located in reverse orientation on chromosome 11 and the same numbering is used in Figure 2. The physical positions are given as the Genome Reference Consortium human genome build 37 coordinates. * Ambulatory 24 h BP responses in GENRES and office BP responses in LIFE. # The positions of TSSs: ACY3-001, 67 418 130, and ACY3-002, 67 415 054. CpG = cytosine–guanine dinucleotide, Chr = chromosome, GRCh37 = the Genome Reference Consortium human genome (build 37), bp = base pair, TSS = transcription start site, Δ = change, SBP = systolic blood pressure, and DBP = diastolic blood pressure.

Since both rs2514036 and its closest methylation sites were associated with BP response to beta blockers, we also analysed their mutual correlations. In both GENRES and LIFE-Fin, the minor rs2514036 G allele was associated with a higher methylation degree of the three methylation sites (CpGs 16–18, see Table 3) with P-values from 5 × 10^−22^ to 1 × 10^−13^ in GENRES and from 2 × 10^−37^ to 7 × 10^−23^ in LIFE-Fin.

## Discussion

In the urgent need for better markers to individualize antihypertensive drug treatment, genetic techniques have been increasingly used for patient profiling, but with only modest success. To our knowledge, the present study represents the first pharmacoepigenomic approach for such purposes. We failed to identify statistically significant genome-wide associations between DNA methylation at CpG sites and BP responses to four different antihypertensive drug classes. However, it was of interest to notice that the extent of methylation at the CpG sites close to the two TSSs of the *ACY3* gene was associated, albeit not at a genome-wide or suggestive significance level, with bisoprolol response, which is in harmony with our previous GWAS studies [5] linking DNA variation at this locus to this drug effect.

The evidence that epigenetic mechanisms indeed contribute to pathophysiology of BP regulation is mostly coming from direct target cell studies in experimental animal models, as well as comparative DNA methylation analyses of circulating blood cells from normotensive and hypertensive patients [29–32]. These studies have recently been extended to human EWASs, which have identified up to approximately 50 sites differentially methylated in normotensive and hypertensive subjects, although none appear to become replicated from a patient cohort to another [33–36]. In the EWAS performed by Richard and colleagues, the most promising methylation sites were located in genes related to vascular phenotypes as well as metabolic ones linked especially to adiposity [34]. In the comparative epigenome-wide association study on BP in two ethnically diverging population groups, Kazmi and colleagues reported four DBP-associating methylation sites [35]. In a recent study by Huang and colleagues, 34 BP-associating CpG sites were cross-validated between two major consortia cohorts and found largely to be located in genes associated with various metabolic phenotypes [36]. Accordingly, the data suggest that at an epigenomic level, BP may be connected with other phenotypes constituting the entity of metabolic syndrome. The DNAs used in these studies [34–36] were isolated from peripheral blood leukocytes; the only exception were the cohorts studied by Richard et al. [34] in which CD4 + T cells were used in less than 10% of the subjects.

We took advantage of two well-phenotyped Finnish patient materials in whom antihypertensive drug responses were determined in a double-blind manner and in whom BP responses were previously associated with genome-wide DNA variations. As our discovery cohort, we used the GENRES Study, a meticulously phenotyped pharmacogenomic study with data from up to four placebo periods and four different antihypertensive drug monotherapy periods. As our replication cohort, we used the Finnish arm of the LIFE Study.

Our top pharmacoepigenomic associations were located in different genes for the various drugs (Table 2). The methylation site cg05560731 in *SNX30* on chromosome 9 showed association with the change in DBP and also suggestively in SBP, exerted by bisoprolol. *SNX30* (Homo sapiens sorting nexin family member 30) is expressed especially in brain and lung tissues. Its genetic variation has been previously found to be associated with increased fasting plasma glucose [37] and, in the GENRES Study, with nocturnal DBP dipping [27]. In our study, cg21740631 in *TENM2* (teneurin transmembrane protein 2) showed association with the change in DBP caused by bisoprolol. *TENM2* is particularly expressed in the heart. Peripheral blood leukocyte methylation changes in *TENM2* have been connected with risk of type 2 diabetes by a recent epigenetic study [38]. Methylation site cg21240861 was the second of the two showing suggestive associations with the change in both DBP and SBP upon hydrochlorothiazide administration in our study. Cg21240861 is located within *DNAJB6* (DnaJ (Hsp40) homolog, subfamily B, member 6) on chromosome 7. In the literature, the gene has been linked to a variety of myopathies [39]. Cg19782883 in *SERTAD4* (SERTAD4 RNA 1) showed association with the change in SBP brought about by losartan. Literature search revealed that *SERTAD4* has appeared mainly in relation to cell studies and has very recently been associated with bone mineral density [40]. Cg14745622 located in *COL9A3* (collagen, type IX, alpha 3) also showed association with the change in SBP caused by losartan. *COL9A3* has previously been studied in relation to hearing loss and different types of aberrations of development, leading to developmental syndromes [41,42]. Some of the methylation sites showing most significant associations with drug effects, including cg14158424 on chromosome 9 and cg14994060 on chromosome 5, were located in intergenic regions.

Pathway analyses for the set of genes identified in EWAS provided some generalized suggestions for the underlying biological pathways. The analyses for bisoprolol resulted in a ‘Renin secretion’ term from KEGG 2021 Human library. According to KEGG 2021 Human library description, the Renin secretion pathway encompasses 69 genes and is responsible for the extracellular fluid volume and BP homoeostasis of the body (https://www.genome.jp/dbget-bin/www_bget?pathway+hsa04924). This finding is consistent with previous results linking plasma renin levels and antihypertensive beta blocker responses. In the GENRES Study, higher pre-treatment plasma renin activity was found to be associated with greater BP responses to bisoprolol [43]. Similar results were discovered between pre-treatment plasma renin activity and BP response to atenolol in the Pharmacogenomic Evaluation of Antihypertensive Responses Study [44]. *ADORA1*, encoding adenosine A1 receptor, was previously linked to SBP in the GWAS study by Evangelou et al [10]. *ITPR3*, encoding inositol 1,4,5-trisphosphate receptor type 3, was previously associated with hypertension in rodent studies [45,46]. In the pathway analysis of bisoprolol responses, *GJC1* was related to the pathway ‘Calcium regulation in the cardiac cell.’ It encodes gap junction protein gamma 1 and was earlier linked to SBP in a GWAS study by Kichev et al [47] and associated with hypertension in rodent studies [48]. Our findings suggest that the regulation of *ADORA1, ITPR3*, and *GJC1* might occur via DNA methylation in SBP or DBP responses to bisoprolol (Table 2). As we lack transcriptomics data and the P-values generally did not survive correction for multiple testing, our results from the pathway analyses need to be interpreted with caution.

In a search for the identification of methylation loci for association with elevated BP per se, Huang and colleagues were able to cross-validate 34 BP-associating CpG sites between two major consortia cohorts [36]. Some of their results replicated in our GENRES analyses at a level of P < 0.05 (see Supplementary Table S2, which shows associations of BP changes with previously cross-validated BP-associated methylation sites). However, none of the top findings of our analyses were amongst these sites. The two CpG sites cg21429551 and cg19390658, located in *GARS* on chromosome 7, were associated with the change in SBP caused by amlodipine (see Supplementary Table S2, which shows associations of BP changes with previously cross-validated BP-associated methylation sites). It is of some interest that cg02711608 in *SLC1A5* on chromosome 19 is associated with the change in SBP caused by both amlodipine and hydrochlorothiazide, thus representing the only methylation site showing association with the effects of more than one antihypertensive drug. Our data thus support the possibility that previously adiposity-associated *SLC1A5* could be an interesting target for further investigation on BP-related methylation changes. We also examined if the main findings of the Kazmi study [35] could be replicated in our analyses. In GENRES, CpG site cg16241714 located on chromosome 8 was associated with the change in both SBP and DBP in the amlodipine group at a significance level of P = 0.008 (data not shown).

In our previous GWAS of the GENRES patients, we demonstrated a significant association with the nucleotide variation at the TSS of the *ACY3* and BP response to bisoprolol [5]. *ACY3*, located on chromosome 11, is a gene coding for aminoacylase 3, an enzyme potentially involved in the biosynthesis of catecholamines through its ability to deacetylate N-acetylphenylalanine and N-acetyltyrosine to phenylalanine and tyrosine, respectively [28]. The gene encodes two different transcripts, ACY3-001 and ACY3-002. The SNP with the strongest association with BP response to bisoprolol was rs2514036, which is located precisely at the TSS of ACY3-002. In the GENRES Study, AG heterozygosity in rs2514036 was found to be associated with an approximately 5 mmHg greater SBP decrease and a 3 mmHg greater DBP decrease in response to bisoprolol compared with homozygote wild-type (AA) subjects |27]. The decrease in BP was even greater in a GG homozygote male. These findings were replicated for BP responses to atenolol in males of LIFE-Fin [17]. The interplay between *ACY3* and beta blocker responses was further substantiated by our previous findings showing that BP response to bisoprolol was correlated with plasma levels of an ACY3 substrate (N-acetylphenylalanine) and end product (phenylalanine) [17].

The findings discussed above prompted us to conduct a closer study on the possible relation between methylation sites close to *ACY3* TSSs and beta blocker responses. As Table 3 and Figure 2 demonstrate, both SBP and DBP responses to bisoprolol were associated, but not at a genome-wide or suggestive significance level, with methylation sites in the promoter areas of both *ACY3* transcripts, ACY3-001 and ACY3-002. These data, supplemented with our previous pharmacogenomic GWAS results, seem to support the role of both *ACY3* genetic and epigenetic variation in BP response to bisoprolol. This assumption is further substantiated by previous findings showing expression of *ACY3* in tissues of potential relevance in BP regulation, such as kidney, liver, heart, brain, neurons, and adrenal medulla [49,50]. In addition, in the Genotype-Tissue Expression (GTEx) database, the rs2514036 minor G allele strongly correlates with lower *ACY3* expression in a variety of tissues (meta-analysis P-value = 5 x 10^−72^) [51] corresponding to its strong association with higher methylation degrees of the three closest methylation sites in the current study.

We acknowledge certain limitations of our study. First, there were only male participants in GENRES, whilst both women and men participated in LIFE. Second, the results of the GENRES Study may be confounded by the carry-over effect from the preceding drug treatment due to a potentially too short subsequent wash-out period. However, longer placebo periods would raise obvious ethical concerns, and our previous studies have validated our study design by demonstrating expected associations between drug effects and plasma renin concentrations [43], as well as showing meaningful pairwise correlations of responses to the four types of antihypertensive drugs [16]. Third, mean 24-hour ambulatory BP measurements were chosen for analyses in GENRES, due to their higher precision and significant correlation with BP office measurements, whilst only office BP measurements were available for study subjects in LIFE. Fourth, whilst we were able to replicate losartan data in GENRES with losartan data in LIFE-Fin, for beta blocker replication studies, we had to use bisoprolol responses in GENRES and atenolol responses in LIFE-Fin. Fifth, we used two different scores for estimation of alcohol consumption in these two studies. Sixth, due to the lack of corresponding gene expression data, we could not assess the functional significance of the identified DNA methylation changes in the CpG sites of the genes. However, we were able to distinguish specific genes, which might be regulated via DNA methylation in SBP and DPB responses to the antihypertensive drugs. Finally, we admit that the use of DNA from other sources than circulating leukocytes, such as from kidney, adrenals, heart, or brain, would possibly constitute a more relevant approach for understanding epigenetic regulation of human BP. However, the strengths of our discovery group (GENRES Study) include its placebo-controlled and cross-over design, thus eliminating the placebo effect of the real drug effects and also providing the assessment of drug specificity of any association noticed.

In conclusion, we did not discover robust associations between DNA methylation sites and BP responses to a beta blocker, a calcium channel blocker, an angiotensin receptor blocker, or a diuretic in two Finnish hypertensive cohorts. However, the findings on the DNA methylation sites close to the *ACY3* TSS may support the role of *ACY3* genetic and epigenetic variation in BP response to bisoprolol.

## Supplementary Material

Supplemental Material

## Data Availability

The data that support the findings of this study are openly available in Mendeley Data (http://dx.doi.org/10.17632/dns8634w2x.1).
