# Supplementary material for: Pharmacoepigenetics of hypertension: genome-wide methylation analysis of responsiveness to four classes of antihypertensive drugs using a double-blind crossover study design
Source: Epigenetics. 2022 Feb 25;17(11):1432–45. doi: 10.1080/15592294.2022.2038418 (PMC9586691; doi:10.1080/15592294.2022.2038418)
Supplement: Supplemental Material [file KEPI_A_2038418_SM7309.zip › supplementary/Nuotio et al_revised Supplementary Tables and Figures.docx]

**Supplementary Figure S1. Flow charts depicting the general design of the GENRES and the LIFE studies.** Arrows indicate occasions of blood pressure measurements. Ate, atenolol; HTZ, hydrochlorothiazide; Losa, losartan; Pre, prestudy period; Plac, placebo.

**
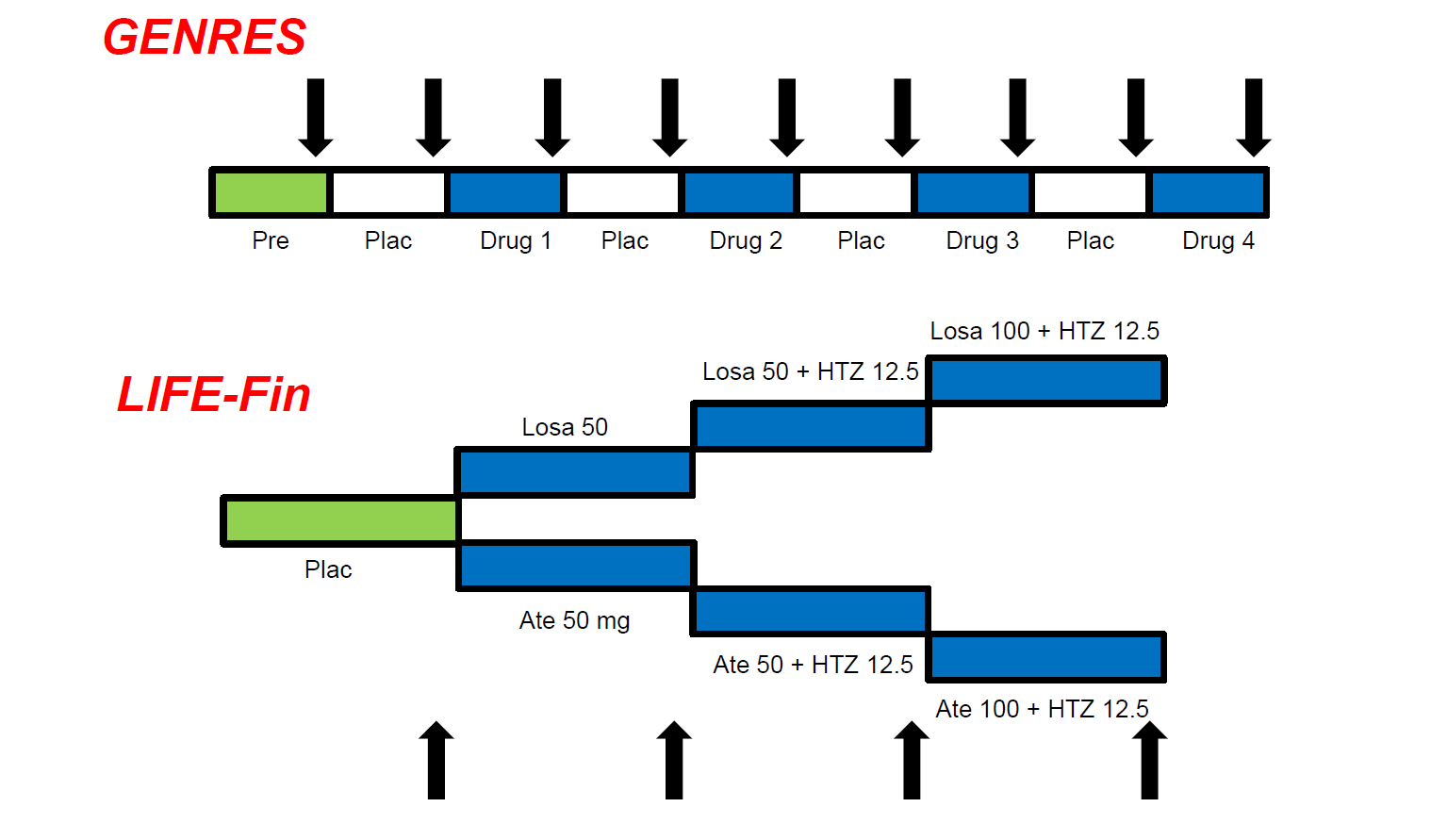
**

**Supplementary Figure S2. QQ plots for regression model results (P-values) in all drug and blood pressure response analyses in GENRES.**

∆SBP

∆DBP

AMLODIPINE


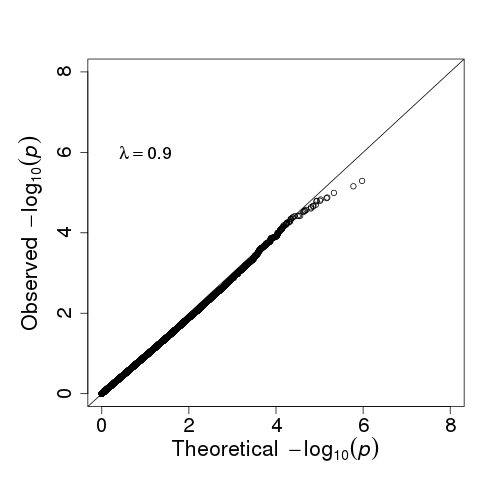

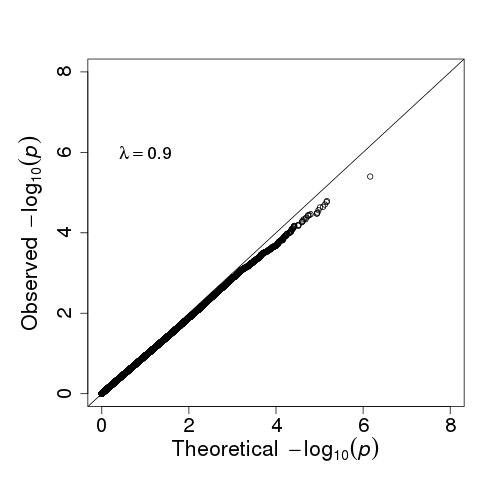


BISOPROLOL


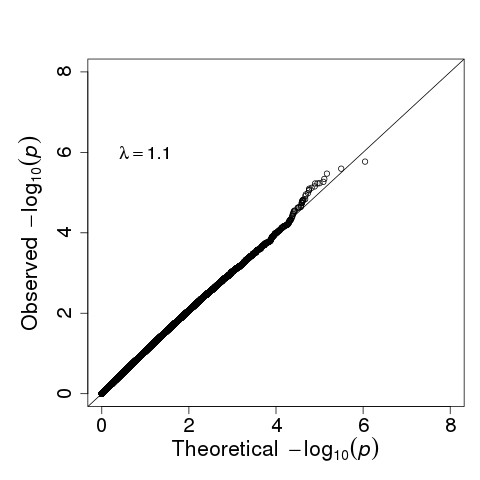

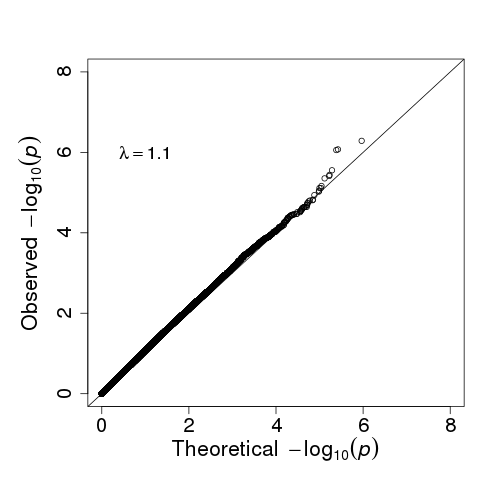


HYDROCHLOROTHIAZIDE


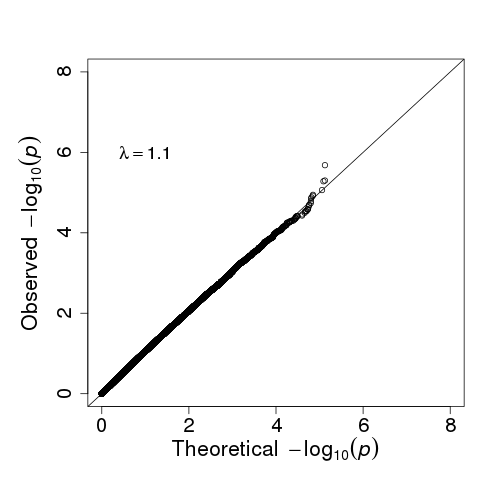

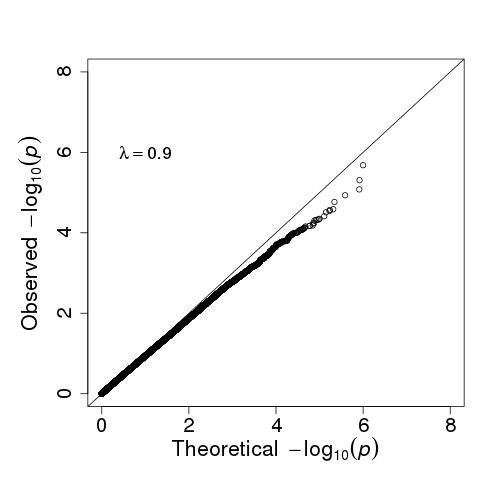


LOSARTAN


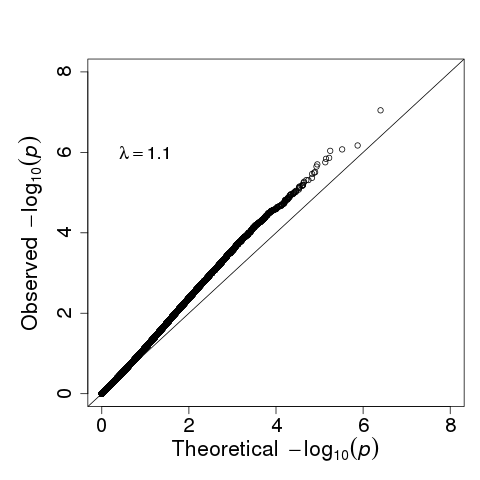

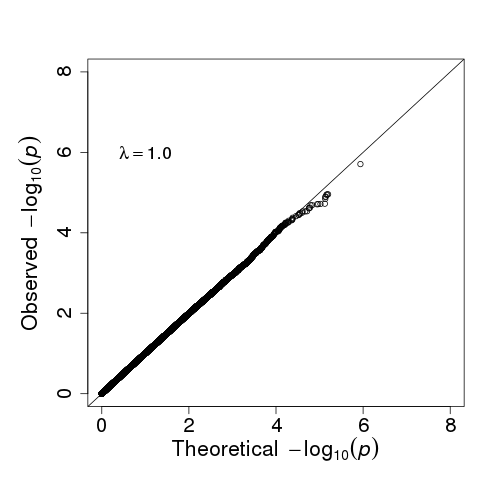


**Supplementary Table S1. An attempt to replicate the most significant EWAS data of GENRES (Table 2) in LIFE**

|  |  |  |  | Δ**SBP** | | Δ**DBP** | |
| --- | --- | --- | --- | --- | --- | --- | --- |
|  | **CpG** | **Chr : position** | **Nearest gene** | **Effect** | **P** | **Effect** | **P** |
| Atenolol | |  |  |  |  |  |  |
|  | cg14158424 | 9 126763957 | *LHX2* | * | * | -0.80 | 0.17 |
|  | cg05560731 | 9: 115 632 737 | *SNX30* | 1.56 | 0.22 | 0.22 | 0.72 |
|  | cg21740631 | 5 : 167 660 070 | *TENM2* | * | * | -0.96 | 0.15 |
|  | cg01938422 | 1 : 35 659 480 | *SFPQ* | 1.42 | 0.19 | * | * |
|  | cg18012642 | 6 : 119 255 579 | *MCM9* | -1.83 | 0.11 | * | * |
|  | cg05347334 | 4 : 2 439 397 | *RP11-503N18.1* | * | * | -0.32 | 0.59 |
|  | cg12667196 | 9 : 108 456 734 | *TMEM38B* | 1.06 | 0.40 | 0.63 | 0.30 |
|  | cg14275626 | 9 : 135 549 588 | *GTF3C4* | * | * | 0.07 | 0.93 |
|  | cg04640885 | 2 : 145 273 345 | *ZEB2* | * | * | 0.36 | 0.54 |
|  | cg23054533 | 8 : 139 095 979 | *RP11-238K6.1* | * | * | -1.83 | 0.0015 |
|  | cg16866321 | 7: 62 153 290 | *RP11-196D18.1* | -1.00 | 0.38 | * | * |
|  | cg04822851 | 1: 203 095 988 | *ADORA1* | -1.12 | 0.25 | * | * |
|  | cg13889422 | 2: 220 492 557 | *SLC4A3* | -1.00 | 0.40 | * | * |
|  | cg19703259 | 16 : 70 612 843 | *IL34* | 0.26 | 0.86 | * | * |
|  | cg07021268 | 19 : 47 921 051 | *MEIS3* | * | * | -0.34 | 0.48 |
|  | cg19755776 | 6 : 29 067 386 | *SERPINB6* | -0.09 | 0.94 | * | * |
|  | cg01074392 | 21 : 44 037 324 | *AP001626.1* | -0.95 | 0.41 | * |  |
|  | cg13097433 | 17 : 42 877 358 | *GJC1* | * | * | 0.21 | 0.73 |
|  | cg11706030 | 2 : 163 225 840 | *GCA; KCNH7* | * | * | -0.38 | 0.55 |
|  | cg06206086 | 1 : 36 412 029 | *AGO3* | 0.57 | 0.64 | * | * |
|  | cg03228312 | 20 : 50 808 336 | *ZFP64* | -0.11 | 0.95 | * | * |
|  | cg21541833 | 2 : 207 507 096 | *AC010731.4* | -1.04 | 0.35 | * | * |
|  | cg22065976 | 6 : 33 589 061 | *ITPR3* | * | * | -0.41 | 0.51 |

Supplementary Table S1 (continued)

|  |  |  |  | Δ**SBP** | | Δ**DBP** | |
| --- | --- | --- | --- | --- | --- | --- | --- |
|  | **CpG** | **Chr : position** | **Nearest gene** | **Effect** | **P** | **Effect** | **P** |
| Losartan | |  |  |  |  |  |  |
|  | cg14496951 | 1 : 156265517 | *GLMP* | 0.02 | 0.99 | * | * |
|  | cg14994060 | 5 : 134376489 | *C5orf66* | 1.92 | 0.19 | * | * |
|  | cg19782883 | 1 : 210406123 | *SERTAD4* | 2.35 | 0.06 | * | * |
|  | cg14745622 | 20 : 61447686 | *COL9A3* | 1.33 | 0.39 | * | * |
|  | cg05014952 | 14 : 35873130 | *NFKBIA* | 1.41 | 0.36 | * | * |
|  | cg25955837 | 6 : 46 620 788 | *SLC25A27; CYP39A1* | 0.58 | 0.69 | * | * |
|  | cg11621667 | 7 : 39662995 | *RALA* | 0.37 | 0.76 | * | * |
|  | cg04640216 | 11 : 63 439 065 | *ATL3* | * | * | -0.53 | 0.34 |
|  | cg00322946 | 10 : 22605631 | *COMMD3* | 0.80 | 0.54 | * | * |
|  | cg00383296 | 4 : 42400551 | *SHISA3* | 2.08 | 0.09 | * | * |
|  | cg08681519 | 22 : 41810229 | *TEF* | 0.36 | 0.75 | * | * |
|  | cg02181494 | 22 : 51066755 | *ARSA* | 1.42 | 0.28 | * | * |
|  | cg27270003 | 4 : 37828093 | *PGM2* | -0.33 | 0.75 | * | * |
|  | cg17712828 | 10 : 94833632 | *CYP26A1* | 0.92 | 0.52 | * | * |
|  | cg01653417 | 17 : 80256028 | *HES7* | 2.11 | 0.17 | * | * |
|  | cg07922719 | 9 : 117150338 | *AKNA* | 1.43 | 0.25 | * | * |
|  | cg12446722 | 1 : 226374380 | *ACBD3* | 0.14 | 0.93 | * | * |
|  | cg20458560 | 6 : 146 283 629 | *SHPRH* | -0.08 | 0.95 | * | * |
|  | cg27204776 | 2 : 203777060 | *CARF;WDR12* | 1.78 | 0.16 | * | * |
|  | cg20250570 | 10 : 1034318 | *GTPBP4* | 0.15 | 0.90 | * | * |
|  | cg15147060 | 3 : 88108213 | *CGGBP1* | 1.03 | 0.48 | * | * |
|  | cg26326168 | 12 : 133405726 | *CHFR; GOLGA3* | 4.44 | 0.0017 | * | * |
|  | cg07485279 | 3 : 31574058 | *STT3B* | 3.17 | 0.0077 | * | * |
|  | cg10014408 | 9 : 139305226 | *PMPCA;SDCCAG3* | 2.07 | 0.03 | * | * |
|  | cg06637893 | 16 : 67700960 | *C16orf86;ENKD1* | 2.54 | 0.07 | * | * |
|  | cg06323912 | 16 : 57481690 | *CIAPIN1;COQ9* | 2.73 | 0.06 | * | * |
|  | cg00876175 | 3 : 179 615 032 | *PEX5L* | 1.49 | 0.15 | * | * |
|  | cg14851297 | 11 : 35965488 | *LDLRAD3* | 3.05 | 0.03 | * | * |
|  | cg10007405 | 13 : 19174773 | *LINC00388* | -0.03 | 0.98 | * | * |
|  | cg04107773 | 20 : 62 273 555 | *STMN3* | -0.01 | 0.996 | * | * |
|  | cg27438067 | 10 : 2 119 638 | *RP11-69C17.2* | 0.75 | 0.44 | * | * |

The physical positions are given as the Genome Reference Consortium human genome build 37 coordinates.

*P-value >10^-5^ in GENRES. CpG = cytosine–guanine dinucleotide, Chr = chromosome, Δ = change, SBP = systolic blood pressure, DBP = diastolic blood pressure**.**
