# Supplementary material for: Pharmacoepigenetics of hypertension: genome-wide methylation analysis of responsiveness to four classes of antihypertensive drugs using a double-blind crossover study design
Source: Epigenetics. 2022 Feb 25;17(11):1432–45. doi: 10.1080/15592294.2022.2038418 (PMC9586691; doi:10.1080/15592294.2022.2038418)
Supplement: Supplemental Material [file KEPI_A_2038418_SM7309.zip › supplementary/Nuotio et al_Supplementary Methods.docx]

**Supplementary Materials** / Nuotio et al.

The R script used in the linear regression (EWAS) analysis:

------------------------------------------------------------------------------

# EWAS with R 3.5.2 in GENRES and LIFE-Fin

# Files used for the analysis

# 1. C1

# - merged phenotype and methylation data

# Normalization methods for non-normally distributed parameters (Blom was used for our analysis)

# https://www.rdocumentation.org/packages/rcompanion/versions/2.3.7/topics/blom

# Mangiafico, S.S. 2016. Summary and Analysis of Extension Program Evaluation in R, version 1.18.8. rcompanion.org/handbook/

blom = function(x, method="general", alpha=pi/8,

complete=FALSE, na.last="keep", na.rm=TRUE,

adjustN=TRUE,

min=1, max=10, ...){

if(complete){x=x[complete.cases(x)]}

Ranks = rank(x, na.last=na.last, ...)

if(adjustN==FALSE){N = length(x)}

if(adjustN==TRUE) {N = sum(complete.cases(x))}

if(method=="blom") {Score = qnorm((Ranks-0.375)/(N+0.25))}

if(method=="vdw") {Score = qnorm((Ranks)/(N+1))}

if(method=="tukey") {Score = qnorm((Ranks-1/3)/(N+1/3))}

if(method=="rankit") {Score = qnorm((Ranks-1/2)/(N))}

if(method=="elfving"){Score = qnorm((Ranks-pi/8)/(N-pi/4+1))}

if(method=="general"){Score = qnorm((Ranks-alpha)/(N-2*alpha+1))}

if(method=="zscore") {Score = (x-mean(x, na.rm=na.rm))/sd(x, na.rm=na.rm)}

if(method=="scale") {

Score = (((x - min(x, na.rm=na.rm)) *

(max - min)) /

(max(x, na.rm=na.rm) - min(x, na.rm=na.rm)) +

min)

}

return(Score)

}

# Normalization of nonnormally distributed principal component 2

pheno$PC2_norm <- blom(pheno$PC2, method = "blom", complete = TRUE)

# Epigenome-wide association analysis

met_norm <- blom(c1[,mbx[i]], method = "blom") #normalization of methylation degrees

results <- lm(c1[,modelname] ~ met_norm + AGE + SMOKING + ALCOHOL + c1[,covname] + CD8T + CD4T + NK + Bcell + Mono + Gran + Array + PC1 + PC2_norm + PC3 + BMI + CREA + as.factor(c1$Slide), data=c1) #For LIFE-Fin SEX was also used as a covariate
